# Cell fusion between tumor cells and macrophages promotes the metastasis of OSCC patient through the activation of the chemokine signaling pathway

**DOI:** 10.1002/cam4.6940

**Published:** 2024-03-08

**Authors:** Chengwan Xia, Qian Zhang, Yumei Pu, Qingang Hu, Yuxin Wang

**Affiliations:** ^1^ Department of Oral and Maxillofacial Trauma Orthognathic Plastic Surgery Nanjing Stomatological Hospital, Medical School of Nanjing University Nanjing China

**Keywords:** cell fusion, chemokine signaling pathway, macrophages, OSCC, tumor metastasis

## Abstract

**Background:**

Tumor metastasis is responsible for the high mortality rate of patients with oral squamous cell carcinoma (OSCC). Although many hypotheses have been proposed to elucidate the mechanism of tumor metastasis, the origin of the metastatic tumor cells remains unclear. In this study, we explored the role of cell fusion in the formation of OSCC metastatic tumor cells.

**Methods:**

Murine OSCC tumor cells and macrophages were fused in vitro, and the cell proliferation, migration, and phagocytosis abilities of hybrid cells and parental cells were compared. Subsequently, we compared the transcriptome differences between hybrid and parental cells.

**Results:**

Murine OSCC tumor cells and macrophages were successfully fused in vitro. The cytological and molecular experimental results revealed that OSCC tumor cells obtained a migration‐related phenotype after fusion with macrophages, and the migration ability of hybrid cells was related to the activation of the “chemokine signal pathway”.

**Conclusion:**

After fusion with macrophages, the chemokine signaling pathway in OSCC tumor cells was activated, leading to metastasis.

## 1 INTRODUCTION

Metastasis of malignant tumors is a major cause of death in cancer patients.^1^ Oral squamous cell carcinoma (OSCC) is the most common malignant tumor in the oral and maxillofacial regions and can metastasize to regional lymph nodes at an early stage,^2^ significantly affecting the long‐term survival and quality of life of patients with OSCC.^1^, ^3^ Although the process of malignant tumor metastasis has been delineated in detail,^4^ the mechanism by which epithelial cells acquire metastasis‐related biological characteristics has not been clearly explained. The theories of “epithelial to mesenchymal transition (EMT)”^5^, ^6^, ^7^, ^8^, ^9^ and “stem cell origin of metastatic tumor cells”^10^, ^11^, ^12^ are the two mainstream hypotheses that explain the metastasis of malignant tumors at the present stage. Nevertheless, many unexplained phenomena still exist for both hypotheses.^4^, ^13^, ^14^, ^15^ For example, epithelial‐derived malignant tumor cells regain epithelial‐associated features (mesenchymal‐epithelial transition [MET]) in distal organs after experiencing loss of epithelial features (epithelial‐mesenchymal transition [EMT]).^4^, ^13^ Moreover, therapeutic approaches based on these two hypotheses have not significantly improved the survival of OSCC patients. Hence, the mechanism of OSCC tumor metastasis requires further study.

Cell fusion refers to the formation of new cells by cytoplasmic or membrane fusion between the same or different cells, which is available for zygote formation and skeletal muscle development.^16^, ^17^ In addition, under physiological conditions, macrophages can improve their phagocytic function through cell fusion to form multinucleated giant cells.^18^ Cell fusion also occurs in many tumor tissues, including breast cancer, ovarian cancer, and malignant melanoma.^19^, ^20^, ^21^, ^22^, ^23^ Studies have shown that by fusing with the surrounding normal epithelial cells, stem cells, and macrophages, tumor cells can rapidly acquire new phenotypes, which not only promote their own malignant progression, but also play an important role in improving their own chemotherapy tolerance. However, whether OSCC cells can obtain a metastatic phenotype through cell fusion remains unclear.

Tumor‐associated macrophages (TAMs) are a vital part of the tumor microenvironment and are closely related to tumor angiogenesis, stromal remodeling, immunosuppression, and metastasis.^24^, ^25^, ^26^ Through careful comparison, we found that the distribution of macrophages in humans is very similar to that in the common metastatic sites of malignant tumors and that the metastatic process of tumors is also similar to the tracking events of macrophages.^27^, ^28^ Given that, Otto Aichel also proposed the hypothesis that tumor cells could acquire the metastatic phenotype by fusion with macrophages in the early 20th century after observing the fusion between tumor cells and macrophages.^29^ Moreover, researchers have found a large amount of evidence of tumor‐macrophage fusion in recent years.^22^, ^30^, ^31^ For example, researchers have detected the presence of the Y chromosome in tumor cells from female patients who had received bone marrow transplants using FISH assay in situ.^22^ Therefore, whether OSCC can acquire metastatic ability by fusing macrophages has attracted our attention.

In this study, murine OSCC cells and macrophages were fused to form tumor‐macrophage hybrids in vitro. Then, the effects of fusion with macrophages on murine OSCC tumor cells at the molecular and cellular function levels have been compared. Finally, RNA sequencing has been performed to uncover the relative molecular mechanisms of OSCC metastasis.

## 2 METHODS

### 2.1 Cell culture

RAW 264.7 murine macrophages and SCC7 murine OSCC tumor cells were obtained from Fudan University (Shanghai, China). RAW 264.7 cells were cultured in DMEM (Gibco, American) supplemented with 10% fetal bovine serum (FBS, Gibco, American) and 1% penicillin/streptomycin (Sigma, American), and SCC7 cells were cultured in RPMI 1640 media (Gibco, USA) supplemented with 10% FBS (Gibco, American) and 1% penicillin/streptomycin. Hybrid cells were cultured in RPMI 1640 medium (Gibco, USA) supplemented with 10% FBS (Gibco, Grand Island, NY, USA) and 1% penicillin/streptomycin. All cells were maintained in a humidified atmosphere at 37°C and 5% CO_2_.

### 2.2 Hybrid cell selection

Before cell fusion, mCherry (anti‐neomycin)‐labeled SCC7 cell lines and GFP (anti‐puromycin)‐labeled RAW 264.7 cell lines were generated by lentiviral infection (Shanghai GeneChem Co., Ltd). Neo‐mCherry‐SCC7 and Puro‐GFP‐RAW 264.7 cells were fused by PEG in vitro. The detailed methods are provided in the supplementary file.

### 2.3 DNA content detection

The DNA content of the hybrid and parental cells was detected using a cell cycle detection kit (Cat. No: KGA511, KeyGEN BioTECN). The detailed methods are provided in the supplementary file.

### 2.4 Immunofluorescence assay

The protein expression of CD163, pan‐CK, Rac2, and CCR4 in SCC7, RAW 264.7, and hybrid cells was detected using a cell immunofluorescence assay. The detailed methods are provided in the supplementary file.

### 2.5 Cell phagocytosis assay

The phagocytosis ability of SCC7, RAW 264.7, and hybrid cells was detected using a phagocytosis assay kit (IgG FITC, 500290–1, Cayman). The detailed methods are provided in the supplementary file.

### 2.6 Cell colony assay

The cell growth patterns of SCC7, RAW 264.7, and hybrid cells were observed by a plate cell colony assay. The detailed methods are provided in the supplementary file.

### 2.7 Cell proliferation assay

The proliferation ability of SCC7, RAW 264.7, and hybrid cells was determined by a CCK‐8 cell proliferation assay using a CCK‐8 detection kit (Beyotime). The detailed methods are provided in the supplementary file.

### 2.8 Cell wound healing assay and transwell cell migration assay

The migration ability of SCC7, RAW 264.7, and hybrid cells was determined by cell wound healing and transwell cell migration assays. The detailed methods are provided in the supplementary file.

### 2.9 Transcriptome sequencing and bioinformatics data analysis

The transcription expression differences between SCC7, RAW 264.7, and hybrid cells have been compared by transcriptome sequencing and bioinformatics data analysis. The detailed methods are provided in the supplementary file. The transcriptome sequencing data could be acquired from GEO database (GSE216480).

### 2.10 CCL22 cytokine chemotaxis assay

The influence of CCL22 on the migration of SCC7, RAW 264.7, and hybrid cells was also determined by transwell cell migration assays. The detailed methods are provided in the supplementary file.

### 2.11 Real‐time PCR (qPCR) assay

Real‐time PCR (qPCR) was performed using SYBR green probes and analyzed using an ABI Prism 7700 sequence detection instrument (Applied Biosystems, Foster City, CA, USA). The detailed methods are provided in the supplementary file. The details of the primers used are provided in Table S1.

### 2.12 Western blot

The protein expression of CCR4, Rac2 in SCC7, RAW 264.7, and hybrid cells were detected by Western blot. The detailed methods are provided in the supplementary file.

### 2.13 Knock down of CCR4

Small interfering RNA (siRNA) oligonucleotide sequences specifically targeting CCR4 (si‐CCR4) and negative control (si‐NC) siRNA were obtained from Shanghai GeneChem Co., Ltd. For transient transfection, the hybrid cells were incubated in 6‐well plates until they reached 50% confluence. Si‐CCR4 and si‐NC at a final concentration of 50 nM were transfected with Lipofectamine®2000 (Invitrogen; Thermo Fisher Scientific, Inc.) according to the manufacturer's protocol. Cells were collected for subsequent experiments at 48 h post‐transfection. The si‐CCR4 sequence was gcTTTCTGTTCAGCACTTGTT.

### 2.14 Animal study

Balb/c athymic nude mice (Male, ∼20 g) were provided by the Comparative Medical Center of Yangzhou University. After the mice were acclimatized to the environment for a week, 1 × 10^6^ SCC7 cells or fusion cells were subcutaneously implanted into the right toe or the tongue of the mice to construct the OSCC mice model. Then, after 3 weeks, the lymph nodes in the right popliteal region or submental region were dissected for further pathological examination.

### 2.15 Immunohistochemical staining (IHC) assay

Immunohistochemical staining (IHC) was used to detect the protein expression of Rac2, CCR4 in OSCC patients. The detailed methods are provided in the supplementary file.

### 2.16 Statistical analysis

Statistical analysis was performed using SPSS (RRID: SCR_002865) and GraphPad Prism (RRID: SCR_002798). One‐way ANOVA was used to evaluate the statistically significant differences between the three groups, and two‐sided Student's *t*‐test was used to evaluate the statistically significant differences between the two groups. A *p*‐value ≤0.05, indicated by asterisks in the figures, was deemed significant.

## 3 RESULTS

### 3.1 Hybrid cell fusion by OSCC tumor cells and macrophage cells in vitro displayed biparental characteristics

**FIGURE 1:**
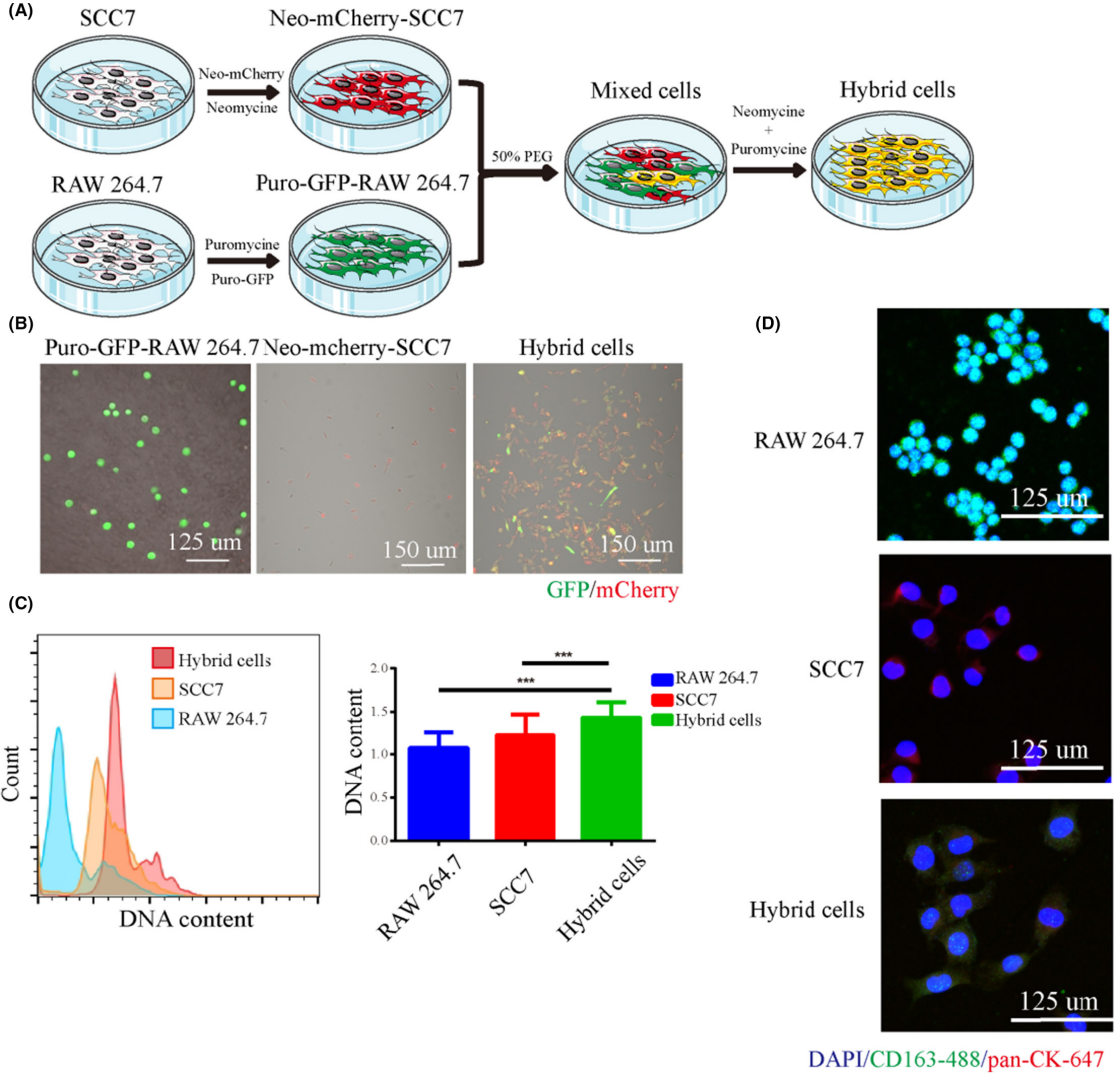
The hybrid cell fusion by SCC7 and RAW 264.7 cells. (A) The diagram of the cell fusion process. (B) Immunofluorescence images of Puro‐GFP‐RAW 264.7, Neo‐mCherry‐SCC7 and hybrid cells. (C) DNA content. (D) Expression of CD163 and pan‐CK in RAW 264.7, SCC7, and hybrid cells. * *p* < 0.05, ** *p* <  0.01, *** *p* < 0.001.

As shown in Figure 1A,B, OSCC tumor SCC7 cells were labeled with Neo‐mCherry, and RAW 264.7 macrophages were labeled with Puro‐GFP. Neo‐mCherry‐SCC7 and Puro‐GFP‐RAW 264.7, cells were fused with PEG to form hybrid cells in vitro and further purified by neomycin and puromycin, as shown in Figure 1A,B. DNA content was also detected, and the results showed that the DNA content of the hybrid cells increased significantly compared to that of the parental cells (Figure 1C). To observe whether the hybrid cells inherited the characteristics of the parental cells, we detected the expression of the OSCC marker pan‐CK and macrophage marker CD163 in the hybrid cells. Immunofluorescence images showed that both pan‐CK and CD163 were expressed in hybrid cells, as shown in Figure 1D. In addition, phagocytosis of RAW 264.7, SCC7, and hybrid cells was detected, and the results showed that the phagocytic ability of OSCC tumor cells was enhanced after fusion with macrophages (Figure S1).

### 3.2 Fusion with macrophage cells promotes the migration of parental SCC7 tumor cells

**FIGURE 2:**
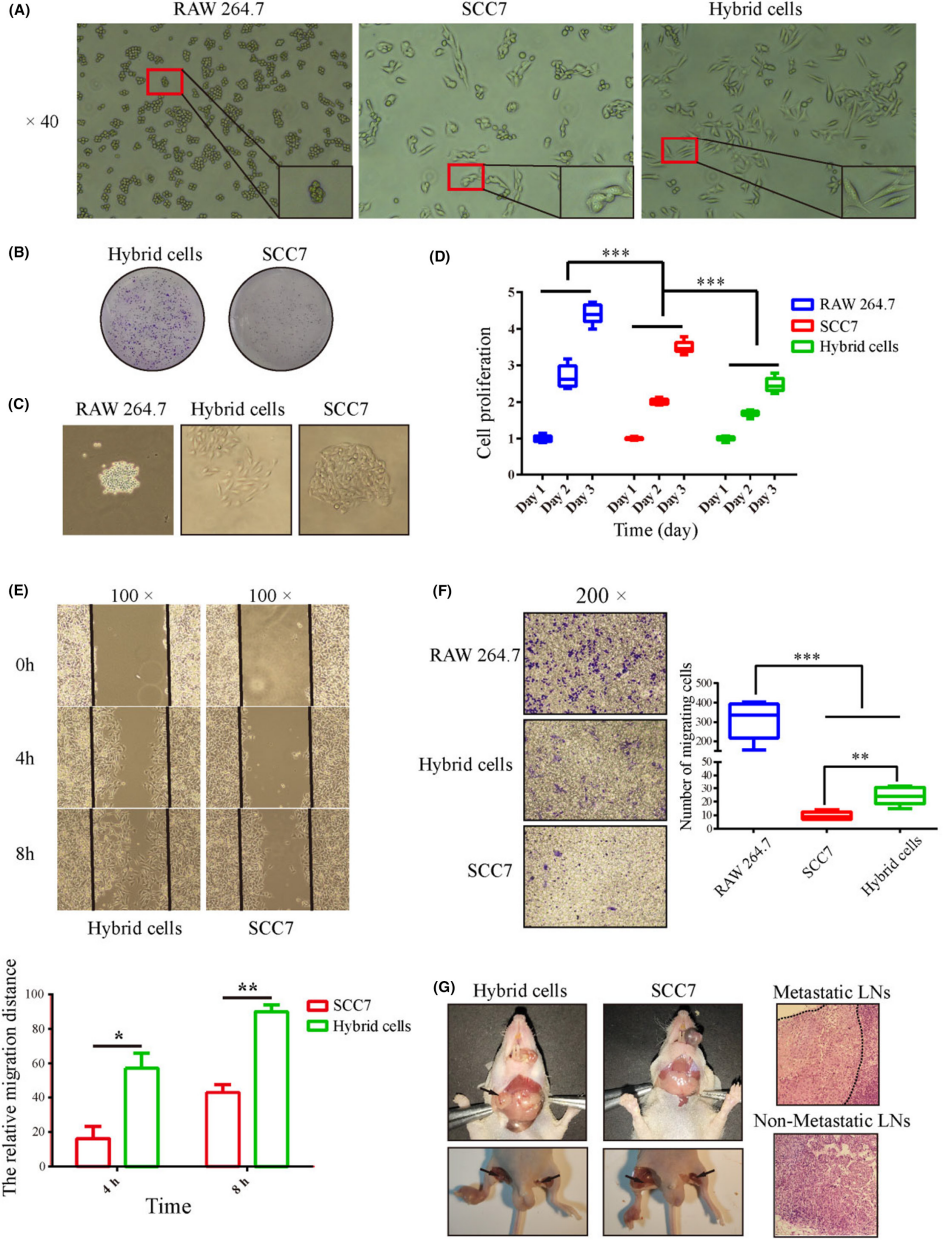
The biological difference between parental SCC7 tumor cells, parental macrophages and hybrid cells. (A) Cell morphologies. (B) The proliferation rates. (C) The colony numbers. (D) The growth patterns. (E, F) The migration abilities. (G) The lymph node metastasis rate of hybrid cells and parental tumor cells. * *p* < 0.05, ** *p* <  0.01, *** *p* < 0.001.

To investigate the effects of macrophage fusion on OSCC tumor cells, we first compared the morphologies of parental SCC7 tumor cells and hybrid cells. The results showed that more cell processes appeared in the hybrid cells, whereas the parental SCC7 tumor cells had a smooth morphology (Figure 2A). We further observed the clonal capacity and cell growth pattern of parental SCC7 tumor cells and hybrid cells using plate cell cloning experiments. The results revealed that the colony number of hybrid cells was higher than that of parental SCC7 tumor cells, and hybrid cells grew more dispersed than the parental SCC7 tumor cells (Figure 2B,C). Moreover, we observed differences in cell proliferation and migration between the hybrid and parental cells. The results showed that compared to parental SCC7 tumor cells, although the proliferation rate of hybrid cells decreased (Figure 2D), their migration ability was obviously enhanced (Figure 2E,F). Further animal studies also revealed that the tumor formed by hybrid cells showed stronger lymph node metastasis ability than parental SCC7 tumor cells (Figure 2G). The lymph node metastasis rates of hybrid cells were 100% (tongue, 4/4) and 83.3% (toe, 5/6), whereas those of SCC7 were both 0% (tongue, 0/3; toe, 0/6).

### 3.3 The chemokine signaling pathway in OSCC tumor cells was enhanced after fusion with macrophages

**FIGURE 3:**
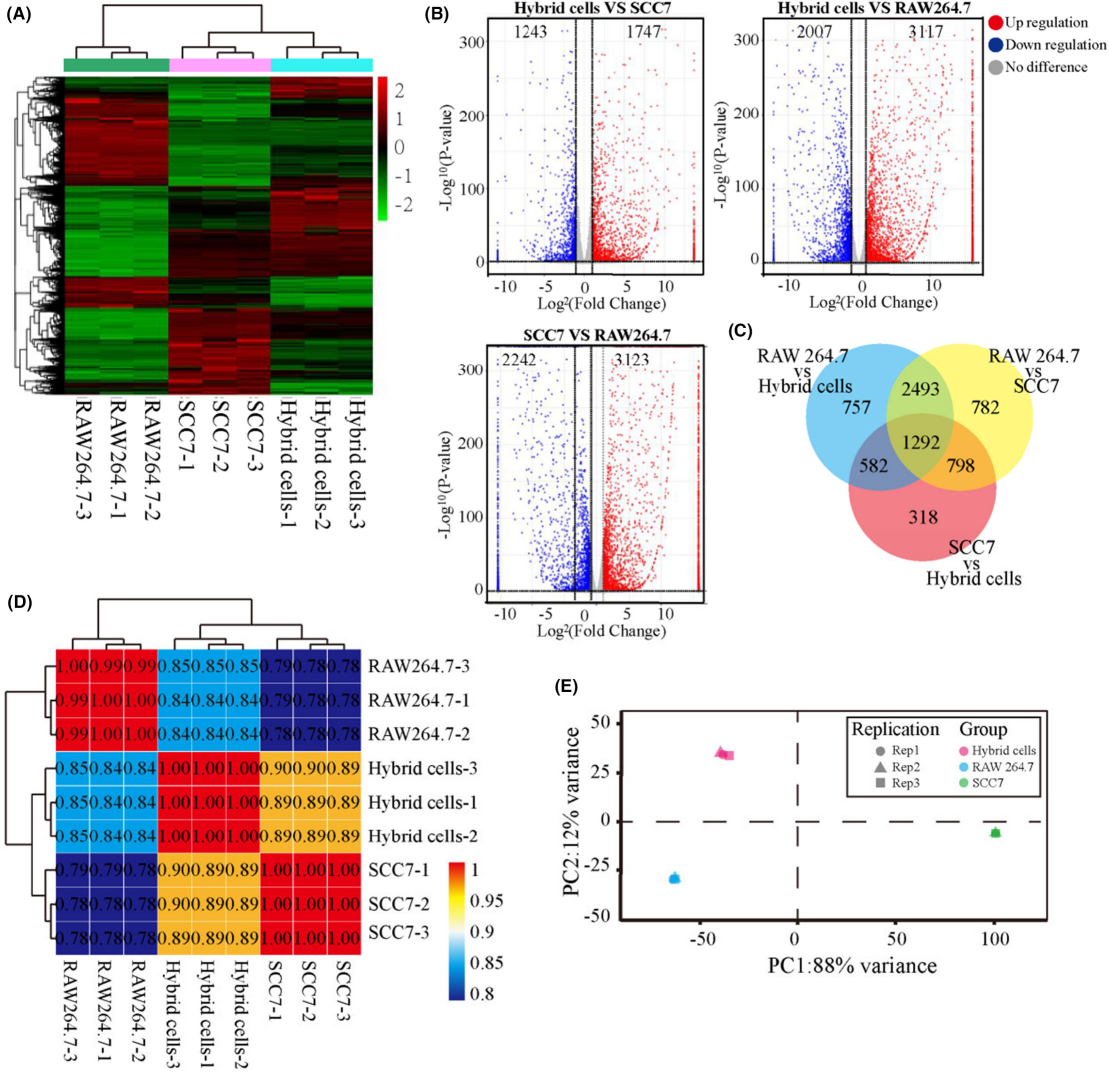
The correlation between hybrid cells and parental cells. (A) Genetic gene map. (B) Volcano map. (C) Venn diagram. (D) Sample correlation analysis. (E) Principal component analysis (PCA) analysis.

To further compare the differences between OSCC tumor cells, macrophage cells, and hybrid cells at the molecular level, we performed transcriptome sequencing on RAW 264.7, SCC7, and hybrid cells. The transcriptomic results showed significant differences at the transcriptional level between the hybrid cells and parental OSCC tumor cells/macrophage cells, as shown in the genetic heat map (Figure 3A), volcano map (Figure 3B), and Venn diagram (Figure 3C). However, according to the sample correlation analysis (Figure 3D) and principal component analysis (PCA) (Figure 3E), the hybrid cells had a strong correlation with the two parental cells at the transcriptional level, which confirmed that OSCC cells could form a new tumor cell subtype by fusion with macrophage cells in vitro.

**FIGURE 4:**
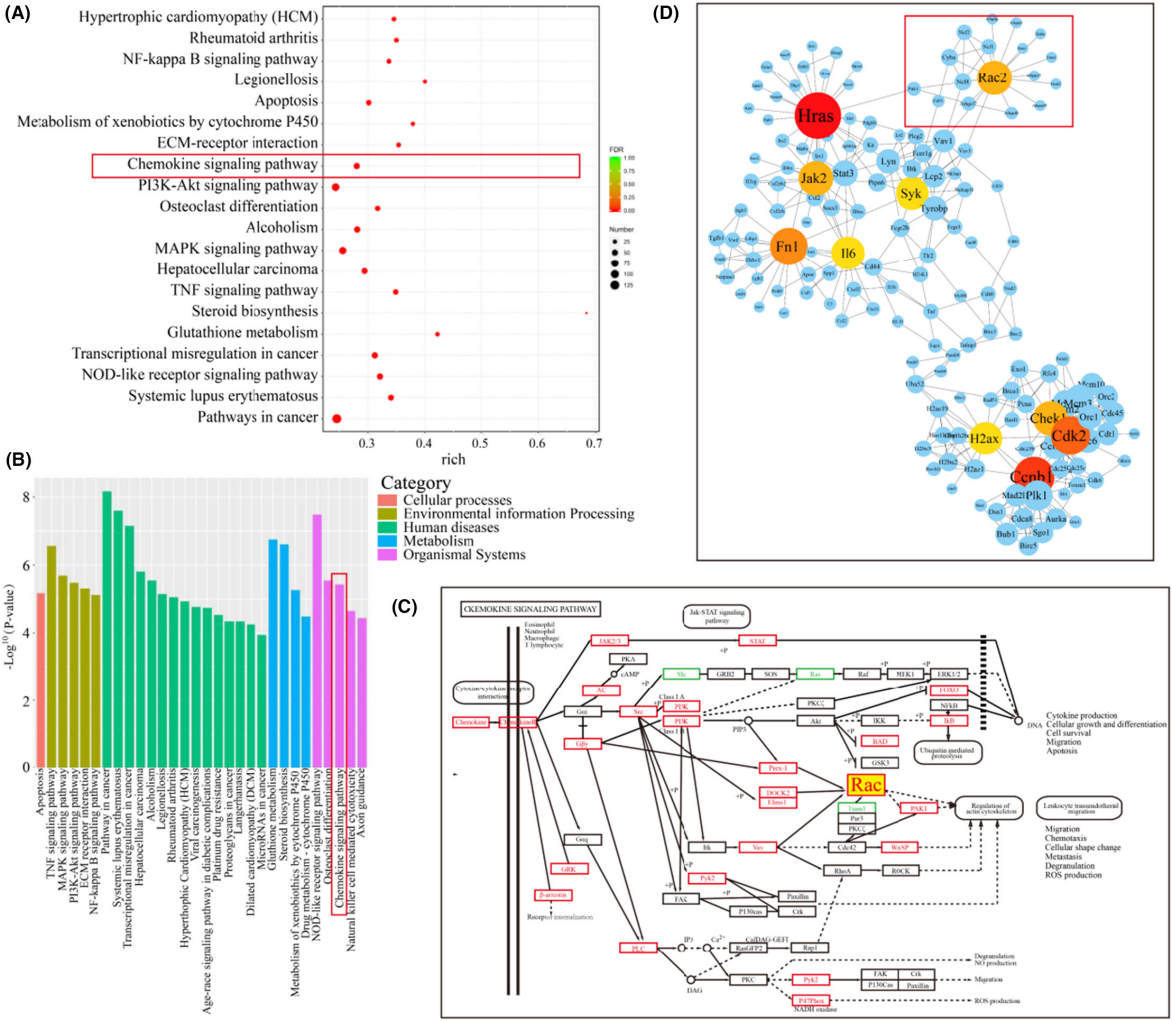
After fusion with parental macrophages cells, the chemokine signaling pathway hybrid cells was enhanced compare to parental SCC7 tumor cells. Bubble diagram (A) and histogram (B) of the GO pathway analysis showed that the chemokine signaling pathway was enhanced in hybrid cells compared with parental tumor cells. (C) KEGG pathway enrichment analysis also showed that the expression of genes in the chemokine signaling pathway was upregulated. (D) PPI analysis showed the top 10 altered genes were HARS, Rac2, Jak2, Fn1, IL6, Syk, Chek1, Cdk2, H2ax, and Ccnb1.

To further explore the effect of fusion with macrophages on OSCC tumor cells at the transcriptional level, the transcriptome data were analyzed by GO analysis (Figure 4A,B) and KEGG pathway enrichment analysis (Figure 4C). These results remind us that compared to the parental SCC7 tumor cells, the chemokine signaling pathway of the hybrid cells was obviously enhanced. Moreover, Rac2, a member of the RAC family, as a core protein in the chemokine signaling pathway, was also listed among the top 10 altered genes in the PPI network, as shown in Figure 4D. According to previous studies, the chemokine signaling pathway is closely related to the biological behaviors of macrophages, including chemotactic migration, metastasis, and cell deformation.^32^, ^33^ This suggests that OSCC tumor cells may acquire relevant abilities by fusion with macrophages to promote their own lymph node metastasis or distant metastasis.

**FIGURE 5:**
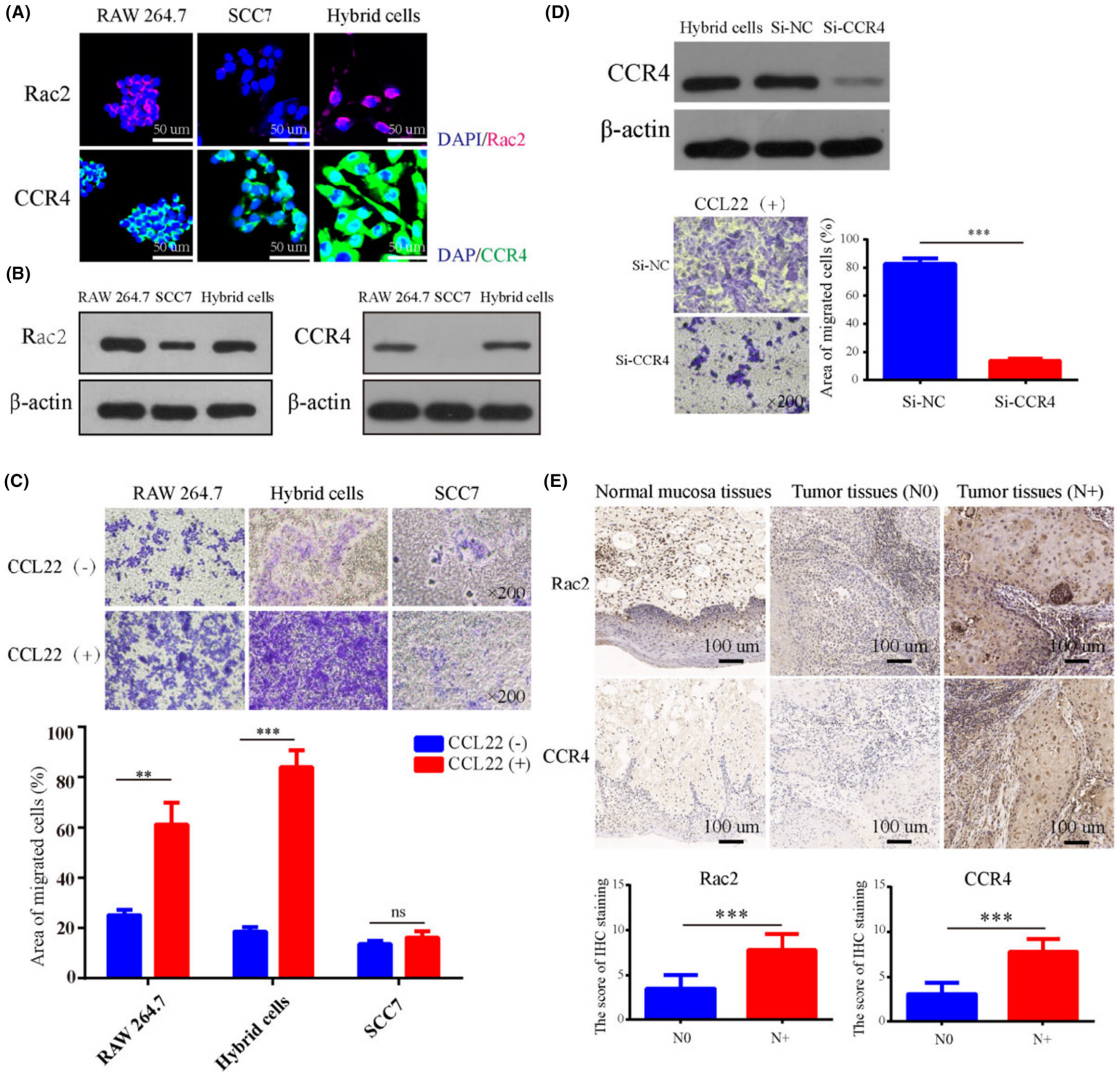
The protein expression of Rac2 and CCR4 in OSCC tumor cells were related to lymph node metastasis. The protein expression of Rac2 and CCR4. (A) Immunofluorescence and (B) Western blot. (C) The effect of CCL22 on the cell migration. (D) The effects of CCR4 knock down on the migration ability of hybrid cells. (E) The expression of Rac2 and CCR4 in OSCC tumor tissues and its correlation with lymph node metastasis. *N* = 76. * *p* < 0.05, ** *p* <  0.01, *** *p* < 0.001.

Subsequently, we further validated the expression levels of signaling molecules in the chemokine signaling pathway by qPCR and found that the expression levels of Rac2, PAK1, DOCK2, Elmo1, Prex1, and Lyn in hybrid cells were enhanced compared to those in parental SCC7 tumor cells (Figure S2A and Table  S2). Moreover, the expression of chemokines and chemokine receptors also increased, with CCR4 being the most upregulated chemokine receptor (Figure S2B,C and Table  S2). Immunofluorescence and Western blot were used to further detect the protein expression of Rac2 and CCR4 in RAW 264.7, SCC7 and hybrid cells. The results showed that after fusion with macrophages, the protein expression of Rac2 and CCR4 also increased in hybrid cells compared to that in parental SCC7 tumor cells (Figure 5A,B). As the ligand of CCR4, CCL22 is rich in lymph nodes,^34^ bones,^35^ lungs,^36^ and brain.^37^ We further observed the effect of CCL22 on the migration of RAW 264.7, SCC7, and hybrid cells. The results also indicated that the hybrid cells showed a stronger CCL22 tendency than the parental SCC7 tumor cells (Figure 5C). Moreover, as shown in Figure 5D, when CCR4 was knocked down, the effect of CCL22 on the migration ability of hybrid cells was suppressed. Furthermore, the expression of Rac2 and CCR4 in clinical OSCC tumor samples was detected by IHC, and the results showed that high expression of Rac2 and CCR4 was related to lymph node metastasis in patients with OSCC (Figure 5D). Further survival analysis also indicated that OSCC patients with high Rac2 expression had shorter disease‐free survival (Figure S3).

### 3.4 Tumor‐macrophage hybrid cells in OSCC tumor cells were related to lymph node metastasis

**FIGURE 6:**
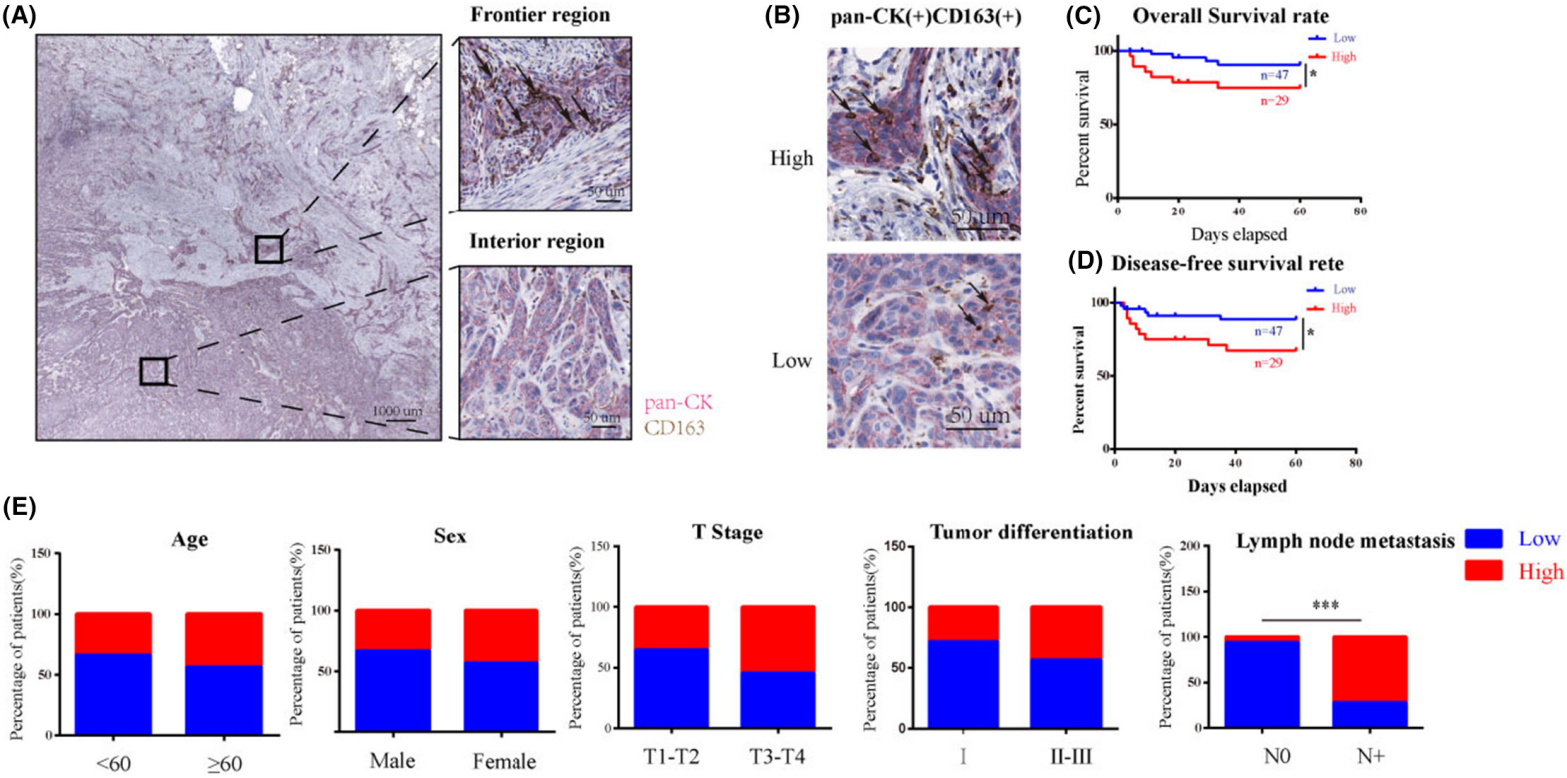
The presence of tumor‐macrophage hybrid cells was related to lymph node metastasis. (A) The spatial distribution of pan‐CK + CD163+ cells in OSCC tumor tissues (the black arrow is pan‐CK + CD163+ cells). (B) Double immunohistochemical staining of pan‐CK and CD163 in OSCC paraffin block samples. *N* = 76. (C, D) The relationship between the number of pan‐CK + CD163+ cells and the prognosis of patients with OSCC. The demarcation between “high” and “low” was established based on whether there were more than 10 hybrid cells observed in a single high‐power field of view. *N* = 76. (E) The relationship between the number of pan‐CK + CD163+ cells and the clinical parameters of patients with OSCC. *N* = 76.

To demonstrate the presence of hybrid cells in OSCC tumor tissues that have the characteristics of both tumor cells and macrophages, we have observed the spatial distribution of hybrid cells in OSCC tumors by double IHC. The images indicated that pan‐CK + CD163+ hybrid cells were mainly distributed at the front of tumor invasion (Figure 6A). Paraffin block samples and clinical parameters of patients with OSCC were also obtained, and the results showed that the presence of pan‐CK + CD163+ hybrid cells was related to lymph node metastasis and poor prognosis (Figure 6B–E).

## 4 DISCUSSION

Metastasis is responsible for the mortality of patients with malignant tumors, including OSCC.^4^ Undoubtedly, elucidating the mechanism of tumor metastasis is essential for improving the prognosis of patients with malignant tumors. The theories of “EMT” and “stem cell origin of metastatic tumor cells” are the mainstream views about the origin of tumor metastasis.^5^, ^6^, ^10^, ^11^ The former believed that the formation of metastatic tumor cells was the result of the gradual accumulation of random mutations and clonal selection in epithelial cells, whereas the latter believed that metastatic tumor cells originated from tissue stem cells. However, neither theory explains how tumor cells acquire biological characteristics associated with metastasis. “Myeloid cell origin of metastasis” was another hypothesis to explain the origin of metastatic tumor cells, which was proposed by the German pathologist Otto Aichel.^29^ This hypothesis states that tumor cells can fuse with macrophages in vivo and acquire the ability to metastasize. Since then, a growing body of research has demonstrated that tumor cells can fuse with normal cells, including macrophages, stem cells, endotheliocytes, and mesenchymal cells, in vivo or in vitro.^20^, ^22^, ^31^, ^38^, ^39^ Moreover, these studies also confirmed that tumor cells could acquire a new biological phenotype, including enhanced proliferative capacity and migration ability by cell fusion. In this study, we demonstrated that OSCC tumor cells can fuse with macrophages in vitro. Through cytological and molecular experiments, we further found that OSCC tumor cells could express a molecular marker of macrophages (CD163), and the migration and phagocytosis abilities of OSCC tumor cells were also significantly enhanced after fusion with macrophages. This finding suggests that OSCC tumor cells can acquire the biological phenotype of macrophages by fusion.

To explore the influence of fusion with macrophages on OSCC tumor cells, we compared the molecular changes in OSCC tumor cells before and after fusion with macrophages at the transcriptional level. Through bioinformatics analysis, we found that the hybrid cells acquired the molecular characteristics of both parental cells. Furthermore, compared to parental OSCC tumor cells, the expression of molecules in the chemokine signaling pathway is significantly enhanced, which is closely related to chemotactic migration and metastasis.^32^, ^33^ As the typical cell types of myeloid immune cells, macrophages are one of the most functional cells in the body. Macrophages can migrate widely to tissues, organs, and vascular systems under the chemotactic action of chemokines. A further review of the literature also revealed that the upregulated expression of chemokine receptors in tumor cells is closely related to the metastasis of tumor cells. Previous studies have reported that high expression of CCR4 in tumor cells is related to lymph node metastasis and distal organ metastasis in malignant tumors, including head and neck, colon, and breast cancers.^34^, ^35^, ^40^, ^41^ In this study, we found that the expression of CCR4 in hybrid cells was significantly higher than that in the parental SCC7 tumor cells. Moreover, using the transwell cell migration assay, we confirmed that CCL22 could enhance the migration ability of hybrid cells. Moreover, retrospective clinical data also showed that CCR4 expression in tumor cells was associated with lymph node metastasis. Thus, the CCR4‐CCL22 signaling axis in hybrid cells plays an important role in tumor metastasis in patients with OSCC.

## 5 CONCLUSION

OSCC murine tumor cells can enhance their migration ability by fusing with macrophages during tumor progression. Activation of the CCR4‐CCL22‐related chemokine signaling pathway after tumor cell‐macrophage cell fusion is an important mechanism. In the future, factors influencing the rate of cell fusion between OSCC tumor cells and macrophages need to be further explored to reduce the metastasis rate in patients with OSCC.

## AUTHOR CONTRIBUTIONS

**Chengwan Xia:** Conceptualization (equal); data curation (lead); formal analysis (lead); methodology (lead); writing – original draft (lead). **Qian Zhang:** Data curation (supporting); funding acquisition (supporting); methodology (supporting); validation (supporting). **Yumei Pu:** Data curation (supporting); methodology (supporting). **Qingang Hu:** Funding acquisition (lead); writing – review and editing (equal). **Yuxin Wang:** Conceptualization (equal); project administration (equal); supervision (equal); writing – review and editing (equal).

## FUNDING INFORMATION

This study was supported by the Nanjing Clinical Research Center for Oral Diseases [2019060009], the Nanjing Department of Health [YKK22177] and Cultivation Program for Junior Talents of Nanjing Stomatological School, Medical School of Nanjing University [0222R207/0222C108].

## CONFLICT OF INTEREST STATEMENT

The authors declare no financial interests.

## ETHICS STATEMENT

The collection and use of patient samples was approved by the Medical Ethics Committee of the Institute Affiliated Stomatology Hospital, Medical School of Nanjing University [NJSH‐2022NL‐15]. Written informed consent was obtained from all patients. All animal experiments were approved by the Care Committee of Nanjing University [IACUC‐D2202062].

## Supporting information


Data S1:

## Data Availability

The datasets generated in the current study are available from the corresponding author upon reasonable request.
