# Supplementary material for: Cell fusion between tumor cells and macrophages promotes the metastasis of OSCC patient through the activation of the chemokine signaling pathway
Source: Cancer Med. 2024 Mar 8;13(4):e6940. doi: 10.1002/cam4.6940 (PMC10923029; doi:10.1002/cam4.6940)
Supplement: Supplementary file 1 — Data S1: [file CAM4-13-e6940-s001.docx]

**Cell fusion between tumor cells and macrophages promotes the metastasis of OSCC patient through the activation of the chemokine signaling pathway**

**Methods**

1. Hybrid cell selection

Specifically, 5×10^5^ Neo-mCherry-SCC7 and 10^5^ Puro-GFP-RAW 264.7 cells were fully mixed and centrifuged at 800 rad for 5 min. The supernatant was then discarded and 500 μL of 50% PEG solution (Sigma, USA) was added dropwise to the cell pellet at 37 $℃$ for 60 s. Subsequently, 9 mL of fresh RPMI 1640 medium was added and the mixture was centrifuged again at 800 rad for 5 min. Finally, the cells were cultured in fresh RPMI 1640 medium supplemented with 10% FBS and 1% penicillin/streptomycin for 24 h to form hybrid cells. After the formation of hybrid cells was confirmed by laser scanning confocal microscopy (Nikon, Japan), neomycin and puromycin were added to the medium to remove the unfused RAW 264.7 and SCC7 cells.

1. DNA content detection

The DNA content of the hybrid and parental cells was detected using a cell cycle detection kit (Cat. No: KGA511, KeyGEN BioTECN). Specifically, hybrid cells, SCC7 and RAW 264.7, were cultured in 6-well plates, washed twice with PBS, and the cell concentration was adjusted to 10^6^/mL. Subsequently, 1 mL of cell suspension was centrifuged and the pellet was resuspended in 70% ice-cold ethanol and fixed at 4 $℃$ overnight. The fixed cells were then washed with PBS again and stained with 500 μL of PI/RNase A at room temperature in the dark for 45 min. Flow cytometry was used to detect red fluorescence at 488 nm.

1. immunofluorescence assay

The protein expression of CD163, pan-CK, Rac2, and CCR4 in SCC7, RAW 264.7, and hybrid cells was detected using a cell immunofluorescence assay. Briefly, 1 × 10^3^ cells (SCC7, RAW 264.7, and hybrid cells) were seeded in glass-bottomed culture dishes. After 24 h, the cells were fixed with a 40% formalin solution for 30 min, permeabilized with 0.1% Triton X-100 for 10 min, and blocked with 3% BSA for 30 min at room temperature. The cells were incubated with primary antibodies against CD163 (Abcam Cat# ab182422, RRID:AB_2753196, USA), pan-CK (Abcam Cat# ab215838, USA), Rac2 (60077-1-Ig, Proteintech, USA), and CCR4 (Abcam Cat# ab216560, USA) overnight at 4$℃$. Dylight-conjugated anti-mouse IgG (647-conjugated anti-mouse IgG, Abcam, USA) and DyLight-conjugated anti-rabbit IgG (488-conjugated anti-rabbit IgG, Abcam, USA) were used as the secondary antibodies. Nuclei were stained with DAPI (Beyotime, China) and observed using a confocal scanning system.

1. Cell phagocytosis assay

First, 5 × 10^3^ SCC7, RAW 264.7, and hybrid cells were seeded in glass-bottomed culture dishes. Twenty-four hours later, the culture medium was replaced with fresh culture medium supplemented with latex bead-rabbit IgG-FITC complex (1:100) and incubated at 37 $℃$ for 2 h. The cells were then washed twice with assay buffer, followed by counterstaining with 100 $\mu$L of DAPI (Beyotime, China) for 15 min at room temperature. After two washes, cells were observed using a confocal scanning system.

1. Cell colony assay

The cell growth patterns of SCC7, RAW 264.7, and hybrid cells were observed by a plate cell colony assay. To do so, 2$\times$ 10^3^ SCC7, RAW 264.7 and hybrid cells were placed in 6-well plates separately and cultured for 7 days. The cell growth patterns were observed by microscopy. The cells were stained with a crystal violet staining solution (Beyotime) to determine the colony number.

1. Cell proliferation assay

The proliferation ability of SCC7, RAW 264.7, and hybrid cells was determined by a CCK-8 cell proliferation assay using a CCK-8 detection kit (Beyotime). Briefly, 1 × 10^3^ cells (SCC7, RAW 264.7 and hybrid cells) were placed in a 96-well plate and cultured in a 5% CO_2_ atmosphere at 37 $℃$ overnight. After 1-3 days of incubation, the culture medium was aspirated, and the treated cells were washed with PBS, followed by the addition of 10 $\mu$L of CCK8 solution to each well and an additional incubation for 2 h at 37 $℃$. The absorbance of each well at 450 nm was measured using an IMark Enzyme Mark instrument (Bio-Rad Inc., USA). Cell proliferation ability was calculated based on the absorbance data.

1. Cell wound healing assay and Transwell cell migration assay

The migration ability of SCC7, RAW 264.7, and hybrid cells was determined by cell wound healing and transwell cell migration assays. For the cell wound healing assay, Ibidi Culture-Insert 2 wells were placed in 6-well plates first, and SCC7 and hybrid cells were then seeded for 24 h to form a 500 μm cell scratch. Next, the culture medium was replaced with a fresh medium without FBS. Phase contrast images were acquired at the time of the scratch and 4 and 8 h later. For the Transwell cell migration assay, 100 μL of SCC7, RAW 264.7, and hybrid cells were seeded in the upper chamber with 10% FBS in the lower chamber. Twenty-four hours later, the cells on the underside of the filter were stained with a crystal violet staining solution (Beyotime), and the cell numbers were counted in five fields by microscopy.

1. Real-time PCR (qPCR) assay

Real-time PCR was performed using SYBR green probes and analyzed using an ABI Prism 7700 sequence detection instrument (Applied Biosystems, Foster City, CA, USA). Total RNA was isolated from SCC7, RAW 264.7, and hybrid cells using an RNeasy Plus Mini Kit (QIAGEN) according to the manufacturer’s protocol. Isolated RNA was reverse-transcribed using the RevertAid First Strand cDNA Synthesis Kit (Thermo Xcalibur, RRID:SCR_014593), followed by real-time PCR (qPCR) using FastStart Universal SYBR Green Master (ROX) (Servicebio) and gene-specific primers.

1. Western Blot

SCC7, RAW 264.7, and hybrid cells were harvested and lysed in RIPA buffer containing 1 mM PMSF (Beyotime). The protein concentration of lysed samples was measured using a NanoDrop One system (Thermo Fisher Scientific). Equal amounts of protein (5 μL per lane) from the samples were loaded and separated by SDS-polyacrylamide gel electrophoresis and transferred to a PVDF membrane (Immobilon, Millipore Corporation, Bedford, MA). For western blotting, the membranes were blocked using Tris-buffered saline containing 5% (w/v) skimmed milk powder and 0.1% (v/v) Tween-20, followed by incubation with anti-CCR4 (1:1000, DF10206, Affinity), Rac2 (1:1000, 60077-1-Ig, Proteintech), and $\beta$-actin (1:1000, GB12001, Servicebio). Signals were detected using an ECL system (Amersham, Piscataway, NJ).

1. Transcriptome sequencing and bioinformatics data analysis

Total RNA from SCC7, RAW 264.7, and hybrid cells (n=3 per group) was isolated using TRIzol Reagent (Invitrogen Life Technologies), after which the concentration, quality, and integrity were determined using a NanoDrop spectrophotometer (Thermo Scientific). RNA sequencing libraries were prepared from 2 μg of total RNA with the following modifications: Ribosomal RNA was removed using an Epicenter Ribo-Zero rRNA Removal Kit (Human/Mouse/Rat). Fragmentation was performed using divalent cations at elevated temperatures in an Illumina proprietary fragmentation buffer. Using RNA as a template and random oligonucleotides as primers, the first strand of cDNA was synthesized and RNase H was used to degrade the RNA strand. In the DNA polymerase I system, dNTP with dUTP were used instead of dTTP as raw material to synthesize the second strand of cDNA. Double-stranded cDNA was purified. Double-end repair was then performed, the "A" base at the 3' end was introduced, and the sequencing adapter was connected. The USER enzyme (NEB, USA) was added to degrade the second strand of cDNA containing U. To select cDNA fragments of the preferred 400-500 bp in length, the library fragments were purified using the AMPure XP system (Beckman Coulter, Beverly, CA, USA). DNA fragments with ligated adaptor molecules at both ends were selectively enriched using the Illumina PCR Primer Cocktail in a 15 cycle PCR. The products were purified (AMPure XP system) and quantified using an Agilent high sensitivity DNA assay on a Bioanalyzer 2100 system (Agilent). The sequencing library was then sequenced on a NovaSeq 6000 platform (Illumina) using Shanghai Personal Biotechnology Cp. Ltd.

Raw data were output in a FASTQ format file, clean data were obtained by removing low-quality reads and adapters from the raw data, and quality control was performed using FastQC. Gene expression analysis was performed using HTSeq (RRIDSCR_005514). Then, according to the quantity of expression, principal component analysis (PCA) of each sample was performed using a procmp function of the R language. Pearson’s correlation coefficient was used to indicate the correlation of gene expression levels between the samples. A Venn diagram was used to show the number of genes that differed between samples. DESeq, RRID: SCR_000154 was used to analyze the differences in gene expression, and |log2 fold change| > 1 and p < 0.05 were selected as the conditions for screening differentially expressed genes (DEGs). The volcano map of DEGs was charted using the ggplots2 software package in R. Cluster analysis of DEGs was performed using the pheatmap software package, R. Furthermore, enrichment analysis of DEGs was conducted using gene ontology (GO) and Kyoto Encyclopedia of Genes and Genomes (KEGG) pathway enrichment analyses. Protein-protein interaction (PPI) analysis was performed using the Search Tool for the Retrieval of Interacting Genes/Proteins (STRING) (http://www.string-db.org/), which identifies interactions between proteins translated from mRNAs.

1. CCL22 cytokine chemotaxis assay

First, 100 μL of SCC7, RAW 264.7, and hybrid cells were seeded in the upper chamber with or without CCL22 in the lower chamber. Twenty-four hours later, the cells on the underside of the filter were stained with a crystal violet staining solution (Beyotime), and the cell numbers were counted in five fields by microscopy.

1. Immunohistochemical staining (IHC) assay

IHC was used to detect the protein expression of Rac2 and CCR4 in OSCC patients. First, 76 paraffin blocks were collected from patients with OSCC in 2014. This study was approved by the Medical Ethics Committee of the Institute Affiliated Stomatology Hospital, Medical School of Nanjing University. In detail, 4 μm sections were prepared and processed using a series of graded xylene and alcohol. For antigen retrieval, sections were heated in a microwave oven with 10 mM citrate buffer solution (pH6) for 10 min. Endogenous peroxidase activity was quenched by incubating sections with 0.3% H_2_O_2_ for 5 min. The sections were then blocked with 3% BSA for 1 h at room temperature. Subsequently, for the staining of Rac2 and CCR4, the sections were incubated with Rac2 (60077-1-Ig, Proteintech, USA) and CCR4 (ab216560, Abcam, USA) overnight at 4 $℃$. Sample sections were then incubated with MaxVisionTM HRO-Polymer anti-Mouse/Rabbit IHC (Kit-5020, Fuzhou Max Vision Biotechnology Development Co. LTD, China) for 2 h at room temperature. The signals were developed using the HRP substrate 3,3’-diaminobenzidine (DAB). Nuclear counterstaining was performed using hematoxylin.

**Results**

Table.S1. List of qPCR primers used in this study

| Gene name | Primer sequence (Forward) | Primer sequence (Reverse) |
| --- | --- | --- |
| GAPDH | TGTCAAGCTCATTTCCTGGTATG | TTATGGGGGTCTGGGATGGA |
| Rac2 | CAGCCTGCTCTAGGGGTGTC | AAGAACGCCACACGGAGAAA |
| Rac1 | CATCCCCACCGTCTTTGACA | GCCCCTGGAGGGTCTATCTT |
| Rac3 | CAAGCTGGACCTCCGTGATG | CAGGTACTTGACGGAACCAATC |
| Pak1 | TCTAAGAAGACCTCCAATAGTCAGA | TCAGACACAGTCTTCACATTCAAA |
| DOCK2 | ATTCTTCAAGACCATGCTGGCTA | GGCCCCTTGAGATCATCTGTC |
| Elmo1 | GGTCTTCCGGCACACTGTC | CACCACCTGCATTACCAAGC |
| Prex1 | ATGTTCACTTGGAGCACGGT | AAGGCCTCAAGGATCTTGGC |
| Lyn | AGCAGTGTGCCCGCAAG | TACATCCCATATTTCTCGCTCG |
| CCR2 | AAGGAGCCATACCTGTAAATGC | AGTATGCCGTGGATGAACTGA |
| CCR4 | ACTTTCAGAAGAGCAAGGCAGC | TCTGTGACCTCTGTGGCATTC |
| CXCR3 | GCCATGTACCTTGAGGTTAGT | GGGAGTCAGAGAAGTCGCTC |
| CXCR5 | TAGGCACCAGCACAAACCTTC | AAGGCCAGTTCCTTGTACAGGTC |
| CX3CR1 | CTTCCCATCTGCTCAGGACCTC | CGCCCAAATAACAGGCCTCA |
| CCL2 | CACTCACCTGCTGCTACTCA | GCTTGGTGACAAAAACTACAGC |
| CCL5 | CCAATCTTGCAGTCGTGTTTGT | GGGGATTACTGAGTGGCATCC |
| CCL7 | TCCCTGGGAAGCTGTTATCTTCA | ACAGCGGTGAGGAATTTTGC |
| CCL9 | CAGGCCGGGCATCATCTTTA | AGTAGCTGGCAGTTCACACC |
| CXCL1 | ACCCAAACCGAAGTCATAGCC | TTGTCAGAAGCCAGCGTTCA |
| CXCL5 | TGCCCTACGGTGGAAGTCAT | AGCTTTCTTTTTGTCACTGCCC |
| CXCL2 | GCCCAGACAGAAGTCATAGCC | TTCTTCCGTTGAGGGACAGC |


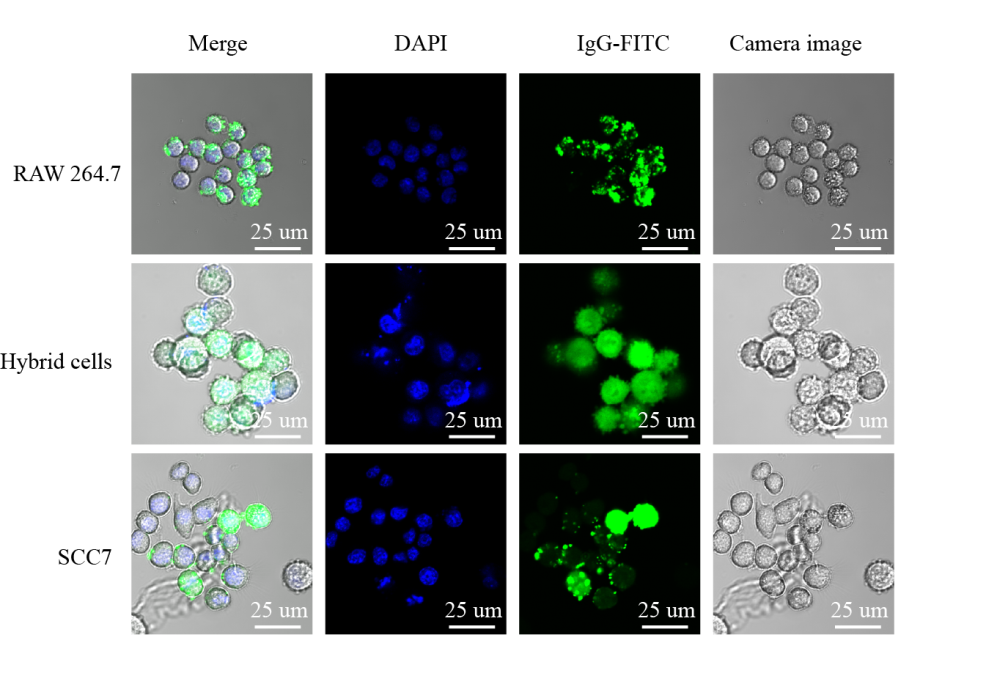


Fig.S1. Phagocytosis ability of RAW 264.7, SCC7 and hybrid cells.

Table.S2. Transcriptome sequencing data of the chemokine signaling pathway.

| Chemokine signaling pathway | | RAW 264.7 | Hybrid cells | SCC7 | P value (Hybrid cells vs SCC7) |
| --- | --- | --- | --- | --- | --- |
| **Rac** | Rac2 | 5375.0±206.8 | 578.7±17.5 | 1.3±0.6 | **<0.001** |
|  | Rac1 | 4616.0±109.7 | 3900.3±74.5 | 5012.0±115.4 | **<0.001** |
|  | Rac3 | 24.7±13.6 | 69.7±4.2 | 88.3±19.0 | 0.172 |
|  | DOCK2 | 16986.0±824.4 | 3340.0±127.6 | 0.3±0.6 | **<0.001** |
|  | Elmo1 | 5878.3±511.5 | 344.3±9.8 | 0.0±0.0 | **<0.001** |
|  | Prex1 | 4587.0±185.6 | 501.0±24.8 | 5.7±4.2 | **0.001** |
| **Src** | Lyn | 4708.7±366.4 | 1861.0±98.6 | 391.7±56.1 | **<0.001** |
|  | Hck | 2454.3±103.9 | 0.3±0.6 | 1.7±0.6 | **0.047** |
|  | Fgr | 372.7±75.5 | 19.0±7.9 | 0.0±0.0 | 0.054 |
|  | Src | 4.7±4.0 | 2553.7±68.9 | 2600.7±36.5 | 0.355 |
|  | Pak1 | 2071.3±64.9 | 41.7±14.3 | 0.0±0.0 | **0.037** |
| **Chemokine receptors** | Cxcr4 | 36.7±3.8 | 0.0±0.0 | 0.0±0.0 | - |
|  | Cxcr5 | 23.0±10.8 | 4.7±4.0 | 1.3±2.3 | 0.283 |
|  | Cxcr3 | 142.3±26.8 | 10.7±9.8 | 7.7±3.2 | 0.641 |
|  | Xcr1 | 7.0±6.2 | 0.0±0.0 | 0.0±0.0 | - |
|  | Cx3cr1 | 7844.0±740.9 | 3.3±4.2 | 1.7±2.1 | 0.569 |
|  | Ccr1 | 7.3±9.5 | 0.0±0.0 | 1.3±1.5 | 0.205 |
|  | Ccr6 | 17.0±11.1 | 0.0±0.0 | 0.0±0.0 | - |
|  | Ccr10 | 1.3±1.5 | 1.3±2.3 | 4.7±4.2 | 0.292 |
|  | Ccr4 | 3.0±5.2 | 33.7±5.1 | 1.7±1.5 | **<0.001** |
|  | Ccr2 | 81.0±8.9 | 3.3±4.9 | 0.0±0.0 | 0.362 |
|  | Ccr5 | 5.3±4.6 | 0.0±0.0 | 0.0±0.0 | - |
| **Chemokines** | Cxcl5 | 0.0±0.0 | 1971.0±713.9 | 0.0±0.0 | **0.009** |
|  | Cxcl3 | 0.0±0.0 | 22.7±16.2 | 0.0±0.0 | **0.072** |
|  | Cxcl1 | 0.0±0.0 | 1636.3±609.8 | 12.7±4.2 | **0.010** |
|  | Cxcl2 | 16.7±16.0 | 740.7±263.7 | 1.7±2.1 | **0.008** |
|  | Ppbp | 0.0±0.0 | 16.0±5.3 | 0.0±0.0 | **0.035** |
|  | Cxcl16 | 465.3±96.7 | 6.3±6.5 | 0.3±0.6 | 0.187 |
|  | Cxcl14 | 203.7±66.0 | 0.0±0.0 | 0.0±0.0 | - |
|  | Cxcl15 | 9.0±2.0 | 46.7±8.1 | 11.3±9.2 | **0.008** |
|  | Cxcl10 | 12.3±5.5 | 74.3±11.7 | 97.7±16.1 | 0.112 |
|  | Pf4 | 2.3±4.0 | 265.0±84.9 | 1.7±1.5 | **0.033** |
|  | Cx3cl1 | 0.0±0.0 | 2.3±1.2 | 48.7±5.8 | **0.004** |
|  | Ccl3 | 231.0±28.8 | 114.3±44.5 | 0.0±0.0 | **0.047** |
|  | Ccl6 | 8.3±5.0 | 5.3±4.5 | 0.3±0.6 | 0.129 |
|  | Ccl4 | 171.7±16.2 | 5.0±5.6 | 0.0±0.0 | 0.195 |
|  | Ccl9 | 0.0±0.0 | 1050.3±217.9 | 2.3±4.0 | **0.014** |
|  | Ccl5 | 0.0±0.0 | 58.0±12.8 | 6.0±2.6 | **0.002** |
|  | Ccl7 | 0.3±0.6 | 15.7±7.5 | 0.0±0.0 | 0.069 |
|  | Ccl2 | 5878.3±511.5 | 25.0±19.1 | 0.0±0.0 | 0.086 |


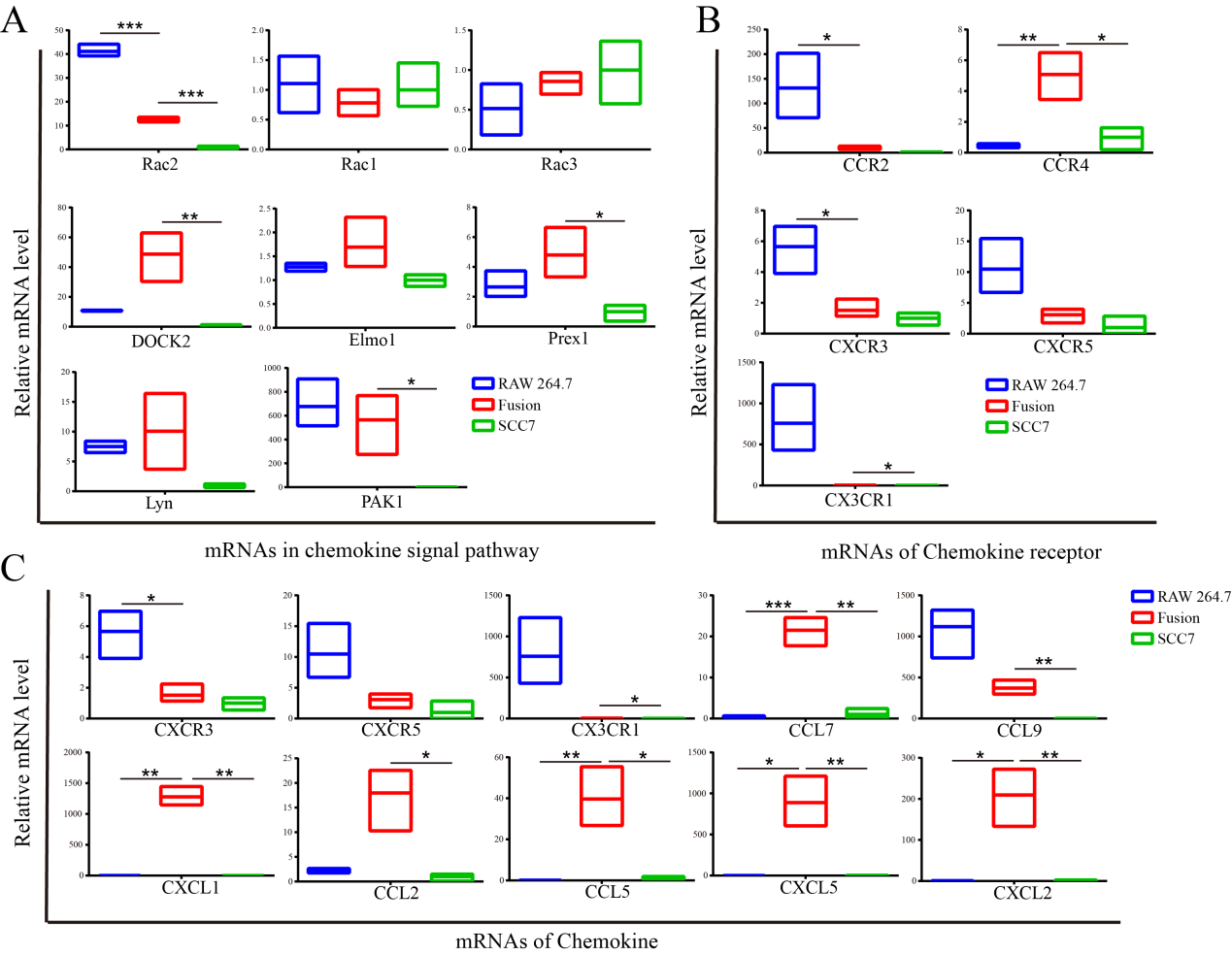


Fig.S2. **Validation of transcription levels of chemokine signaling pathway-related molecules.** (A) The expression level of mRNAs in the chemokine signaling pathway. (B) The expression level of chemokine receptor mRNAs. (C) The expression level of chemokine mRNAs.


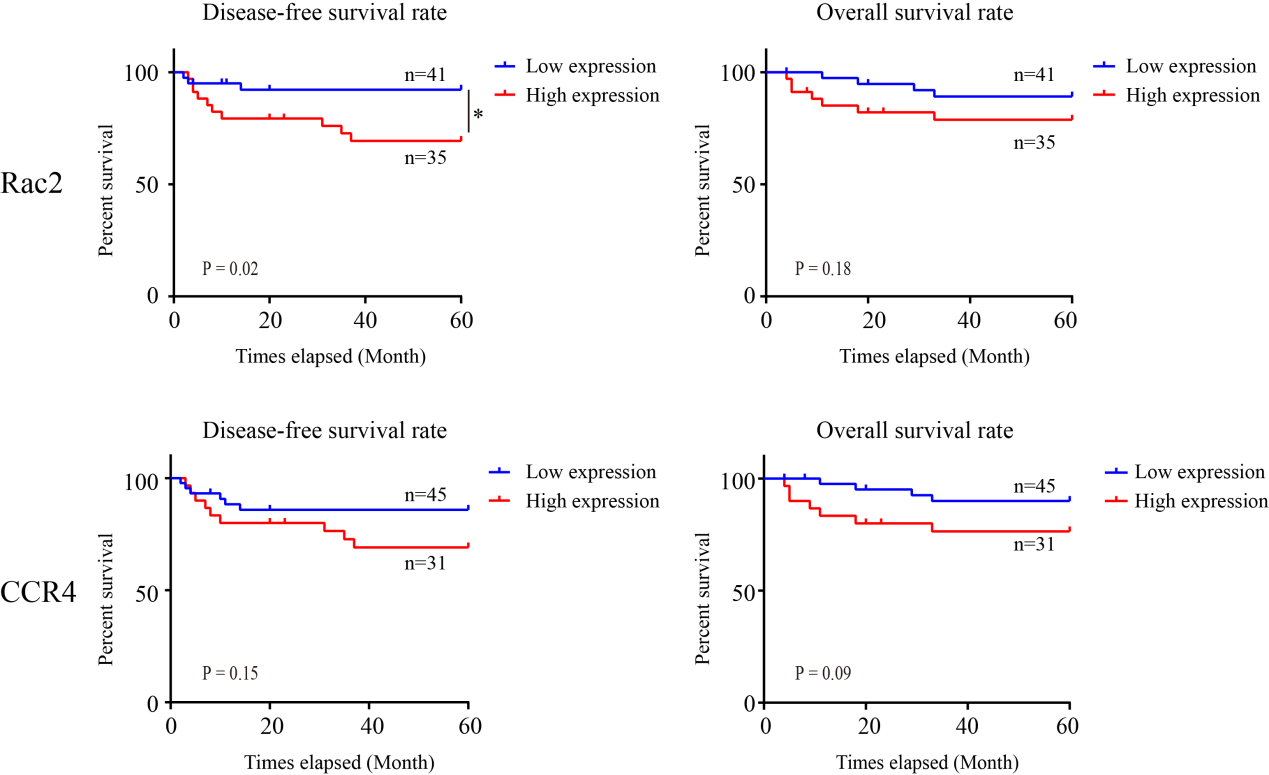


Fig.S3. Relationship between the expression of Rac2 and CCR4 in tumor tissues and the prognosis of patients with OSCC.
